# Isolated fallopian tube torsion with partial hydrosalpinx in a premenarcheal girl: a case report

**DOI:** 10.1186/1752-1947-8-197

**Published:** 2014-06-17

**Authors:** Stjepan Višnjić, Rok Kralj, Božidar Župančić

**Affiliations:** 1 Pediatric Surgery Department, Children’s Hospital Zagreb, Klaićeva 16, Zagreb, Croatia

**Keywords:** Conservative, Hydrosalpinx, Neosalpingostomy, Premenarcheal

## Abstract

**Introduction:**

Isolated fallopian tube torsion as a complication of a preexisting hydrosalpinx is a rare finding in pediatric patients. The obvious rarity of this condition, its subtle diagnostic features and dissonant previous reporting about the appropriate therapeutic approach according to age, future conception capacity and potential complications of possible pregnancies make the decision about which surgical approach to use very difficult. In this report, we describe the case of a patient with such a presentation and review the literature. Very few similar reports of neosalpingostomy in pediatric patients have been published to date.

**Case presentation:**

In our present report, we describe the case of an 11-year-old Caucasian prepubertal girl who presented to our hospital with complaints of abdominal pain in the right lower quadrant, nausea and vomiting. The diagnostic workup led us to conclude that she had a torsion of the right ovary, which was cystically altered. Exploratory surgery revealed a partial hydrosalpinx and consecutive isolated torsion of the fimbrial part. The proximal isthmic part of the fallopian tube was intact and vital. Restorative surgery was performed to create a neosalpingostomy on the viable isthmic part of the tube and remove the cystic and twisted fimbrial and infundibular parts.

**Conclusion:**

The surgical procedure described in this report is technically simple and feasible, but leaves doubts about the final outcome.

## Introduction

Fallopian tube torsion has an estimated prevalence of 1 in 1.5 million women [1]. It generally occurs as adnexal torsion caused by concomitant ovarian pathology. Isolated tubal torsion is a rare event in women of reproductive age and even more so in adolescent girls. It is a rare entity that is a surgical emergency of the utmost importance. Progressive symptoms of an acute abdominal event, although unspecific, often indicate the need for emergency surgery. Recently published papers in which the authors have advocated for restorative surgery oppose directly the traditional approach of salpingectomy. The goals of surgery in these patients is to avoid the risk of suboptimal treatment to achieve a fine balance between maintaining conception capacity and avoiding serious complications of misconception. In our present case report, we describe the technical details of neosalpingostomy as well as recently reported advantages and pitfalls of restorative surgery.

## Case presentation

An 11-year-old prepubertal Caucasian girl was admitted to our emergency room with complaints of abdominal pain in the right lower quadrant, as well as nausea and vomiting. Her symptoms had started 7 days prior to her visit, but her pain had intensified the evening before she presented to our emergency room. She described her pain as moderate and colicky in nature.

**Figure 1:**
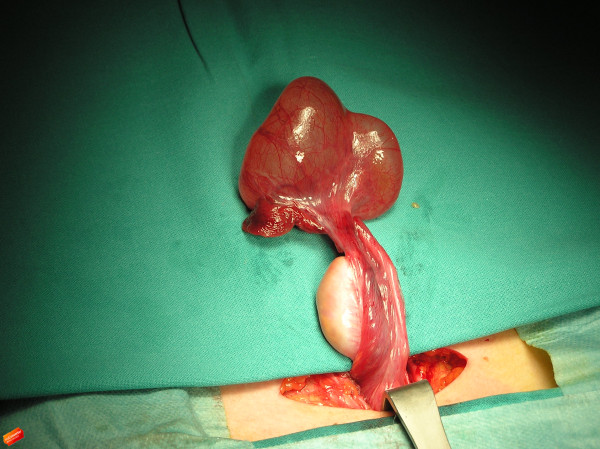
**Post-operative photograph of the convoluted cystic mass of the hydrosalpinx, elongated, viable fallopian tube.** Detorsion was performed through a low transverse Pfannenstiel incision, and a normal ovary was the initial finding.

**Figure 2:**
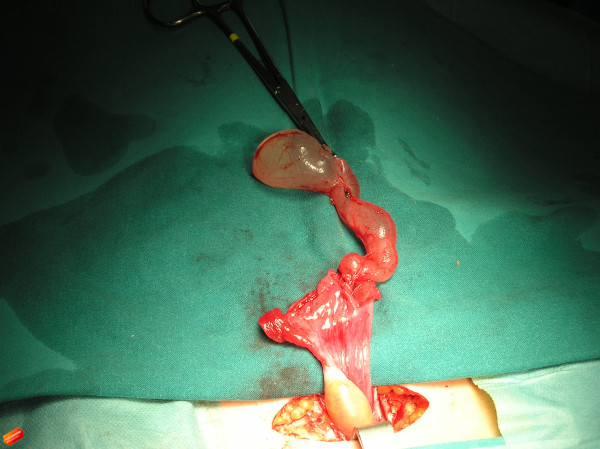
**Post-operative photograph of the debulked, untwisted cystic mass.** We found a cystically altered terminal left fallopian tube.

**Figure 3:**
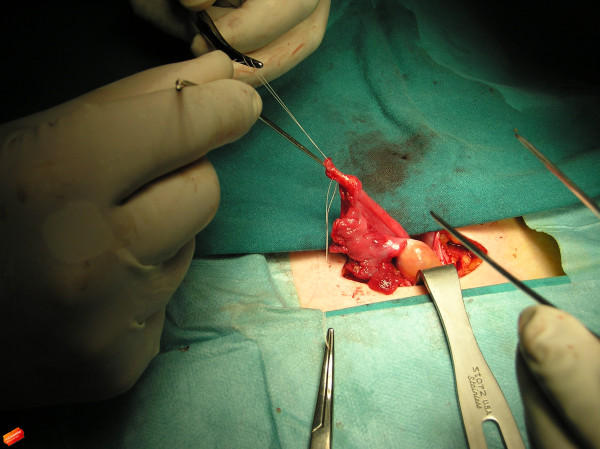
Peri-operative photograph shows a stainless steel probe used for the conductivity check of the tube remnant.

**Figure 4:**
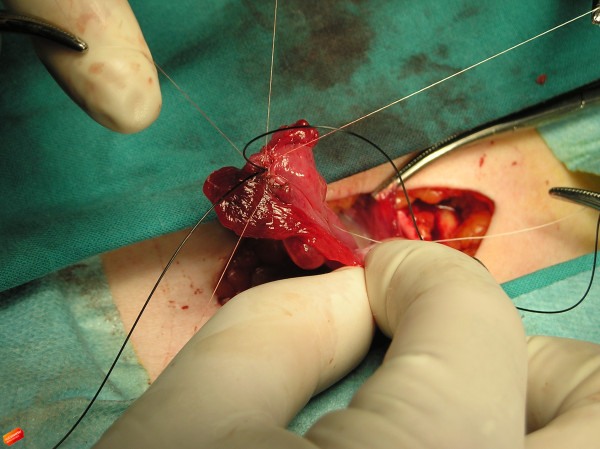
Peri-operative photograph shows nylon suture used to keep the tube orifice open and radially placed nylon sutures for creation of the neostomy.

Upon presentation, she was found to be afebrile, and her laboratory work-up revealed that her white blood cell count was 11,500/ml with 84% neutrophils. The other laboratory examination findings were within the reference ranges. During her physical examination, we detected right lower-quadrant pain without abdominal guarding. An ultrasound examination revealed a hypoechogenic cyst in the right side of the pouch of Douglas measuring 9cm in diameter. Doppler ultrasonography revealed blood flow on the edges of the structure and absence in the central part. The finding was misinterpreted as torsion of the cystically altered right ovary.After a Pfannenstiel incision was made, surgical findings revealed that the uterus, right and left round ligaments, ovaries, cecum and appendix were all normal. The right fallopian tube was twisted and large, distended, cystic, convoluted and filled with fluid that was tapering as it approached the ampular tube. The remaining proximal portion of the tube appeared viable. The contralateral fallopian tube was normal. After we performed a meticulous detorsion (Figure 1), the viable part of the fallopian tube and the cystic, convoluted portion of the hydrosalpinx were clearly exposed (Figure 2). We resected the cystically altered fimbrial part of the fallopian tube with a stainless steel probe and assessed the conductivity of the remnant, which consisted of the ampullary and isthmic parts of the tube (Figure 3), and then we placed a nylon suture which anchored the tube orifice and thus maintained its permeability. Subsequently, we created a neostomy with five radially placed 6-0 Vicryl sutures (Figure 4). The right ovary, uterus and left adnexa were left unchanged.

The pathologic findings confirmed that the excised structure was a cystically altered fallopian tube with a thinned wall and preserved parietal cells. The post-operative period was uneventful. The patient was discharged on the third post-operative day. She is in being followed closely in regular monthly post-period intervals by our gynecologist according to the protocol at our institution.

## Discussion

In our review of the literature, we found reports of 45 cases of pediatric isolated fallopian tube torsion. The mean age of the patients at presentation was 13.2 years [2].

Several risk factors for tubal torsion in adults have been identified: pelvic inflammatory disease, prior pelvic surgery, previous ectopic pregnancy, endometriosis and paratubal cysts [1]. Currently, there are no clearly identified risk factors for the development of isolated torsion of the fallopian tube in the pediatric population, but the authors of a recent report suggested that a congenital malformation of the tube in the peri-pubertal age could be a significant risk factor [3].

Hydrosalpinx is a very rare finding in adolescent girls without a history of pelvic inflammatory disease, and its exact pathogenesis is not clear. Sporadic cases of non-inflammatory hydrosalpinx have been reported as post-surgical complications or as complications of peritoneal drains [4].

Although the presumed course of events in our patient is that hydrosalpinx provoked torsion of the fallopian tube, the possibility that hydrosalpinx was a consequence rather than the cause of the torsion, which could have been provoked by a pre-existing fimbrial cyst, must also be considered. However, unclear etiology should have no impact on therapeutic decision-making in the clinical setting of fallopian tube torsion. The pathology report in our present case confirmed that the excised structure was indeed a cystically altered fallopian tube.

The onset of symptoms in patients such as ours is acute 60% of the time. Peritoneal signs are present in one-third of the patients, but other symptoms are non-specific [5]. Diagnostic imaging (ultrasound and computed tomography) reveal a cystic mass, but rarely differentiate the origin of the mass [6]. The patient’s clinical status will determine the amount and type of pre-operative checkup as well as the urgency of surgery. Most commonly, the diagnosis is made during exploratory surgery.

Few therapeutic options for isolated tubal torsion have been reported to date. Complete or partial salpingectomy is indicated in cases of ischemic, irreversible changes of the tubal wall. Laparoscopic tubal clip occlusion has been described as an alternative to salpingectomy [7]. Radical excision with concomitant tubal pathology, suspected neoplastic changes or hemorrhage is a reasonable choice. A conservative approach such as preservative surgery—either detorsion or partial resection—is another option.

In pediatric patients, the future conception capacity of the patient should always be taken into account. Therefore, partial tubal salvage and neosalpingostomy creation are especially important issues in treating adolescent girls. It is acknowledged that salvage of the ovary is a safe choice in the pediatric population, regardless of how necrotic the ovary appears to be [8,9]. The same philosophy can be applied to tubal salvage after a preliminary evaluation of tubal status [3].

Neosalpingostomy in premenarchal girls with fallopian tube torsion has been reported recently [3,10]. Boukaidi *et al.* proposed a two-stage procedure in which the first step is detorsion of the torqued fallopian tube with puncture of the hydrosalpinx for cytologic and bacteriologic analysis to rule out malignancy and infectious disease. Neosalpingostomy is performed in the second step if the tube is judged to be salvageable after salpingoscopic evaluation. Although we did not perform a salpingoscopic evaluation of the fallopian tube in our patient, the macroscopic appearance of the proximal ampullary and isthmic parts of the tube was favorable, which encouraged us to proceed with neosalpingostomy.

Restorative surgery raises two major concerns. How functional will the fallopian tube be after preservation surgery? Does the potential benefit outweigh the risks of infection and ectopic pregnancy?

Spontaneous conception and successful *in vitro* fertilization (IVF) after tube-preserving surgery are controversial issues. Fair chances of spontaneous conception, as well as more successful IVF-assisted pregnancies, have been reported after a conservative surgical approach as opposed to salpingectomy [11]. Histological examination could help to determine the viability of the tube remnant and resection plane [12]. However, serious concerns remain that a restored tube could expose the patient to a high, unnecessary risk of ectopic pregnancy [12]. According to Bayrak *et al.*, functional repair of hydrosalpinx in adult patients treated for infertility by laparoscopic neosalpingostomy is not recommended because of the dismal spontaneous pregnancy rate and an up to 70% incidence of recurrence of hydrosalpinx, which requires a second surgery before IVF [13].

Does operative fixation of the tube prevent recurrent torsion? Salpingopexy seems reasonable but could change the normal anatomy of the pelvis, either by moving the adnexa outside the pelvis or by distorting the close relationship between the ovary and the fimbriated portion of the tube. Shortening of the mesosalpinx to reduce the tubal mobility may also impair the blood supply to the adjacent ovary [12].

## Conclusion

Isolated torsion of the fallopian tube is a rare clinical entity in the pediatric age group, but pediatric surgeons should nevertheless be aware that it may be the cause of acute onset of abdominal pain. In spite of the availability of advanced imaging techniques, it can be difficult to diagnose isolated tubal torsion on the basis of clinical and imaging grounds alone. Many authors have concluded that the diagnosis can be made only during exploratory surgery.

Therapeutic options should be considered on a case–by-case basis. Although each of the methods has its own advantages and limitations, the choice of management still depends on the patient’s condition and the surgeon’s experience. Bearing in mind all of the controversies about the conservative approach, restoration surgery seems like a fair option.

Neosalpingostomy is technically simple if a significant part of the fallopian tube is vital and conductible. However, clear data about the final impact of this type of surgery on the preservation of the patient’s reproductive potential is still missing. Close long-term follow-up should be maintained in order to avoid complications.

## Consent

Written informed consent was obtained from the patient’s legal guardians for publication of this case report and any accompanying images. A copy of the written consent is available for review by the Editor-in-Chief of this journal.

## Competing interests

The authors declare that they have no competing interests.

## Authors’ contributions

SV performed the surgery, was involved in the follow-up, reviewed the literature and participated in writing the manuscript. RK reviewed the literature and participated in writing the manuscript. BŽ participated in the design of the manuscript and drafted the manuscript. All authors read and approved the final manuscript.
